# A bimodal theranostic nanodelivery system based on [graphene oxide-chlorogenic acid-gadolinium/gold] nanoparticles

**DOI:** 10.1371/journal.pone.0200760

**Published:** 2018-07-25

**Authors:** Muhammad Sani Usman, Mohd Zobir Hussein, Sharida Fakurazi, Mas Jaffri Masarudin, Fathinul Fikri Ahmad Saad

**Affiliations:** 1 Materials Synthesis and Characterization Laboratory, Institute of Advanced Technology (ITMA), Universiti Putra Malaysia, Serdang, Selangor, Malaysia; 2 Department of Human Anatomy, Faculty of Medicine and Health Sciences, Universiti Putra Malaysia, Serdang, Selangor, Malaysia; 3 Department of Cell & Molecular Biology, Faculty of Biotechnology and Biomolecular Sciences, Universiti Putra Malaysia, Serdang, Selangor, Malaysia; 4 Centre for Diagnostic and Nuclear Imaging, Faculty of Medicine and Health Sciences, Universiti Putra Malaysia, Serdang, Selangor, Malaysia; Institute of Materials Science, GERMANY

## Abstract

We have synthesized a bimodal theranostic nanodelivery system (BIT) that is based on graphene oxide (GO) and composed of a natural chemotherapeutic agent, chlorogenic acid (CA) used as the anticancer agent, while gadolinium (Gd) and gold nanoparticles (AuNPs) were used as contrast agents for magnetic resonance imaging (MRI) modality. The CA and Gd guest agents were simultaneously loaded on the GO nanolayers using chemical interactions, such as hydrogen bonding and π–π non-covalent interactions to form GOGCA nanocomposite. Subsequently, the AuNPs were doped on the surface of the GOGCA by means of electrostatic interactions, which resulted in the BIT. The physico–chemical studies of the BIT affirmed its successful development. The X-ray diffractograms (XRD) collected of the various stages of BIT synthesis showed the successive development of the hybrid system, while 90% of the chlorogenic acid was released in phosphate buffer solution (PBS) at pH 4.8. This was further reaffirmed by the *in vitro* evaluations, which showed stunted HepG2 cancer cells growth against the above 90% cell growth in the control cells. A reverse case was recorded for the 3T3 normal cells. Further, the acquired T1-weighted image of the BIT doped samples obtained from the MRI indicated contrast enhancement in comparison with the plain Gd and water references. The abovementioned results portray our BIT as a promising future chemotherapeutic for anticancer treatment with diagnostic modalities.

## Introduction

Graphene is a carbon-based nanomaterial with a two dimensional layered structure. Since the discovery of the nanomaterial by Geim group in 2004 [1], graphene as well as other carbon-based nanomaterials, such as carbon nanotubes (CNT) have been utilized for various research purposes [2, 3] particularly in nanoscience and nanotechnology. It has exceptional properties, which include but not limited to thermal, electrical [4], mechanical [5] and biomedical properties [6, 7].

As a nanomaterial, graphene has high surface area to volume ratio and has two major derivatives, which differ slightly in the functional groups and structures with the graphene nanosheet. Graphene oxide (GO) is one of the graphene derivatives commonly obtained by chemical oxidation of graphite powder via the Hummer’s protocol. During the chemical oxidation process, hydroxyl (OH) and uncharged epoxide (O) groups are introduced into the graphene-like structure [8]. The structural changes due to the addition of these groups make GO more compatible for conjugation with external compounds, such as polymers, and therapeutic agents. The hydrophilicity due to these functional groups makes GO soluble in colloids [9, 10]. According to literature, GO is still the most utilized graphene-based material in drug delivery, especially in anticancer applications [7]. Moreover, the anticancer agents are more likely to be conjugated through hydrogen bonding and π–π non-covalent interactions, which could occur at the GO plane and surface [11]. Another graphene derivative is reduced graphene oxide (rGO), which is derived by thermal, chemical, infrared (IR) or ultraviolet (UV) reduction of GO [12]. It has more similarity with graphene nanosheets in terms of functional groups while it is structurally similar to GO with a vacant −O space on the plane, with a very high surface area. Graphene-based materials vary in surface chemistry and dimensions which is based on the number of layers [13]. They can be categorized into either single layer or bi-layer/multilayer, depending on their orientation.

As mentioned earlier, GO has more tendencies for biomedical applications. It has been used in various anticancer drug delivery applications. Lately, GO is being used in bimodal theranostic delivery systems (BIT). A system that encompasses both therapeutic and contrast or diagnostic agents for intended use in cancer therapy and diagnosis, respectively. It also offers the possibility of anticancer efficacy improvement, contrast enhancement as well as general toxicity reduction [14].

The imaging modalities employed in cancer research or treatment, such as magnetic resonance imaging (MRI) and computed tomography (CT), often require contrast agents due to poor visibilities in body tissues [5]. Contrast agents such as paramagnetic gadolinium chelates are the only FDA approved contrasts for diagnosis with MRI, while iobitridol is used mostly for computed tomography (CT), which are often administered to subjects prior to the test. Some of these contrasts have being reported to have certain toxicities to humans [15]. The gadolinium (Gd)-based contrast boosts the MRI spin–lattice relaxation, T1 and spin–spin relaxation, T2 signals intensities [16], by shortening their relaxation times. Gd-based contrast have previously been used in a BIT setting as the diagnostic component, which were reported to have higher T1 and T2 values than in their pure forms [17]. However, for CT modality in BIT, AuNPs are often used as the diagnostic components. They have also been reported to have higher contrast when compared with the commercial iobitridol contrast [18], due to their high surface area to volume ratio.

Nonetheless, the theranostic approach of cancer research is very much at its early stage and there is much to be done in the area. Only a number of works have reported theranostic systems of cancer treatment and even fewer using GO-based systems [18]. In this work, GO nanosheet was used as nanocarrier in a BIT setting, and a natural occurring compound, chlorogenic acid (CA) was used as therapeutic component. CA is a phenolic compound found mostly in coffee. It has been reported to have a range of properties, including anticancer [19]. The Gd and AuNPs were both used in this work as contrast agents for the MR imaging modality. To the best of our knowledge, no article has reported the above listed combination for simultaneous MRI modality and drug delivery. However, Usman *et al*., 2017 [20], has reported the combination of both agents for solitary use as MRI contrast enhancer, where layered double hydroxide (LDH) nanolayers were used as nanocarrier in a theranostic delivery setting (TDS) [20]. Likewise, no article has reported the use of chlorogenic acid as therapeutic agent in a BIT. Nevertheless, a few articles have conjugated anticancer agents with GO nanosheets [21], by exploiting the ‒OH bonding and π‒π stacking interactions. Similar concept was applied in this work, although no functionalizing agents were used in our work.

## Materials and methods

### Materials

Chlorogenic acid (MW: 354.31 g/mol, PP: 98%), ortho-phosphoric acid (PP: 85%), potassium permanganate (PP: 99%), sulphuric acid (PP: 98%), graphite flakes (100 mesh size), hydrogen peroxide (PP: 35%) and phosphate-buffered saline (PBS) were procured from Sigma-Aldrich (St Louis, MO). Ethyl alcohol (PP: 99.7%) was obtained from Hayman. Gadolinium (III) nitrate hexahydrate (PP: 99.9%) and tetrachloroauric (III) acid trihydrate (PP: 49% Au ─ 393.83 g/mol) were supplied by Acros Organics (NJ USA). Diethyl ether (PP: 85%) and hydrochloric acid (PP: 37%) were provided by Friedemann Schmidt (Parkwood, WA, USA). Human liver hepatocellular carcinoma (HepG2) and normal fibroblast (3T3) cell lines were provided by the American Tissue Culture Collection (ATCC) (Manassas, USA). All experiments were conducted using deionized water (DI).

### Characterization

The pristine and the synthesized nanocomposites were characterized using various techniques, starting with X-ray diffraction which was done on an XRD-6000 diffractometer, Shimadzu, Tokyo, Japan, using CuKα radiation (λ = 1.5406 Å, 40 kV and 30 mA) and scan rate of 0.5°θ/min. Thermo Nicolet model Nicolet 6700 was used for fourier transformed infrared spectroscopy (FTIR) using a KBr disc. Raman spectroscopy analysis was done using a UHTS 300 Raman spectrometer (WITec, Germany) at 532 nm laser excitation wavelength. The drug release study was done on a PerkinElmer ultraviolet−visible spectrophotometer (Lambda35) (PerkinElmer, Boston, MA). TGA/DSC 1HT model (METTLER TOLEDO, Shah Alam, Selangor, Malaysia) was used for thermogravimetric analysis (TGA)/differential thermogravimetric (DTG) analyses, at 10°C/min heating rate and 50 mL/min nitrogen flow rate. Perkin-Elmer spectrophotometer (Model Optima2000DV) was used for inductively coupled plasma atomic emission spectrometry (ICP−ES), CHNS−932 LECO was used for Carbon, hydrogen, nitrogen and sulphur (CHNS) analysis and energy dispersive X-ray spectroscopy (EDS) (FEI Company) were used for compositional studies. Tecnai TF20 X−Twin (FEI, USA) high resolution transmission electron microscope (HRTEM) was used for morphology and structure studies of the pure GO and the nanohybrids.

### Graphene oxide synthesis

The graphene oxide nanocarrier (GO) was prepared via improved Hummer's method as reported previously [8]. A mixture of 360 and 40 mL of H_2_SO_4_ and H_3_PO_4_, respectively was added to the graphite powder (3 g). The mixture was homogenized by stirring after which KMnO_4_ (18 g) was slowly introduced. A rise in temperature (around 50°C) was observed due to the KMnO_4_ addition. Sequel to 12 h of stirring under dark conditions, the mixture was poured into a beaker containing 400 mL iced DI water. 3 mL of H_2_O_2_ was added to the mixture. An immediate change in colour to yellowish-brown was observed. The suspension obtained was filtered/washed via centrifuge using DI water, HCl, ethanol and diethyl ether 200 mL each successively. The suspension was finally filtered and dried at 40°C in a vacuum.

### Graphene oxide/gadolinium and chlorogenic acid nanocomposite

The Gd and chlorogenic acid were individually loaded into the GO nanosheet. At first, the chlorogenic acid (0.6 g) was dissolved in DI water and 0.0008M of gadolinium nitrate was added to the drug solution. 0.2 g of GO powder was then added to the solution. A grey suspension was obtained at low pH. The suspension pH was raised to 5.5 using 0.5 NaOH. The dispersion was stirred overnight in a dark room at 25°C. The suspension was then centrifuged and the slurry recovered was washed 3 times with DI water. The sample was vacuum dried in an oven at 40°C. The nanocomposite is named GOGCA.

### Adsorption of gold nanoparticles on the graphene oxide/gadolinium and chlorogenic acid nanocomposite

The BIT was acquired by adsorption of gold nanoparticle on the GOGCA nanocomposite. The protocol was adopted by modification of the adsorption method reported in [20]. 0.15 g of GOGCA was dispersed in 90 mL of aqueous medium by means of ultra-sonication. Under nitrogen flow, 2% HAuCl_4_ (6 mL) was added to the dispersion and stirred at room temperature. Subsequently, 0.25 M NaOH (2 mL) was introduced into the mixture and stirred for 24 hours at 60°C. The mixture was re-dispersed in DI water (30 mL). Prior to that, the suspension was centrifuged and the liquid was discarded. 20 mL of 1M NaBH_4_ reducing agent was added under stirring to obtain the AuNPs. The slurry was collected via centrifuge and washed repeatedly. The sample was dried at 70°C for 24 hours in a vacuum oven.

### Drug loading and release studies

An ultraviolet-visible spectrophotometer (UV‒Vis Perkin Elmer Inc., Waltham, MA, USA) was employed in the drug loading and release, by way of absorbance and Lambda determination. The Lambda max of the chlorogenic acid (λ_max_ = 364 nm) and calibration curve were determined with the UV-Vis. Prior to that, 25 mg of GOGCA was dispersed in 30 mL of PBS (pH 7.4 and 4.8, respectively). The dispersions were positioned in a 37°C oil bath shaker, and lightly shaken for the drug to release. 3 mL each of the release media were extracted and replaced at different time intervals with PBS until the all the drug was released. The absorbances of the extracted release solutions were determined and used for the drug release estimation.

### Cytotoxicity study

#### Cell culture

RPMI 1640 culture medium (Invitrogen, NZ) was used for culturing the standard fibroblast (3T3) and carcinoma (HepG2) cell lines, which were used for the anticancer and cytotoxicity studies, respectively. The cell lines were cultured at 1% antibiotics (penicillin/streptomycin) and 10% fetal bovine serum (FBV) medium. The cells were maintained in an incubator and humidified chamber. The temperature was set at 37°C in the presence of 5% CO_2_ atmosphere. Subsequently, trypsinization was used in the culture harvest.

#### Cytotoxicity evaluation

The toxicity evaluation was done after the culture harvest. 96‒wells were first plated with the grown cells using 100 μL of cell culture medium. The density of the cells was maintained at 1.0 × 105 per well. The cells were kept for 24 hours to allow attachment to occur. The pure chlorogenic acid, the pure GO and the developed BIT were added at different concentrations. The cells were then allowed another 72 hours for incubation. 5 mg of MTT (3-[4, 5-dimethylthiazol-2-yl]-2, 5-diphenyltetrazolium bromide) was dissolved in 2 mL PBS and added to the 96‒well plates, which resulted in Formazan product after incubation at 37°C. DMSO (100 μL) was introduced into the cells after the cells were briefly shaken. The optical densities of the cells were then measured at 570 nm. The cell viabilities were converted to percentages and the untreated cells were used as references. The experiments and measurements were done in triplicate for accuracy. Only the mean ± standard deviations were considered.

### Imaging studies

The developed BIT was subjected to contrast enhancement test using 3.0 T MRI clinical instrument (3.0 T Siemens Magnetom). The BIT was distributed into different ${Gd}^{3+}\;$concentrations in an aqueous medium that is 2.0, 0.5 and 0.2 *w/v*, respectively. 0.5 *w/v* of Gd(NO_3_)_3_ and DI water were used as reference. The BIT samples were placed in the magnetic source location of the MRI clinical instrument by means of MRI phantom holder. TR/TE: (83/9000) 224*220s and field of view (FOV): 120*120 conditions were maintained all through the acquiring process of the T1‒weighted images. MRI software (Syngo MR E11, Siemens, Erlangen, Germany, 2013 was used for the analysis of the acquired T1‒weighted images.

## Results and discussion

**Fig 1:**
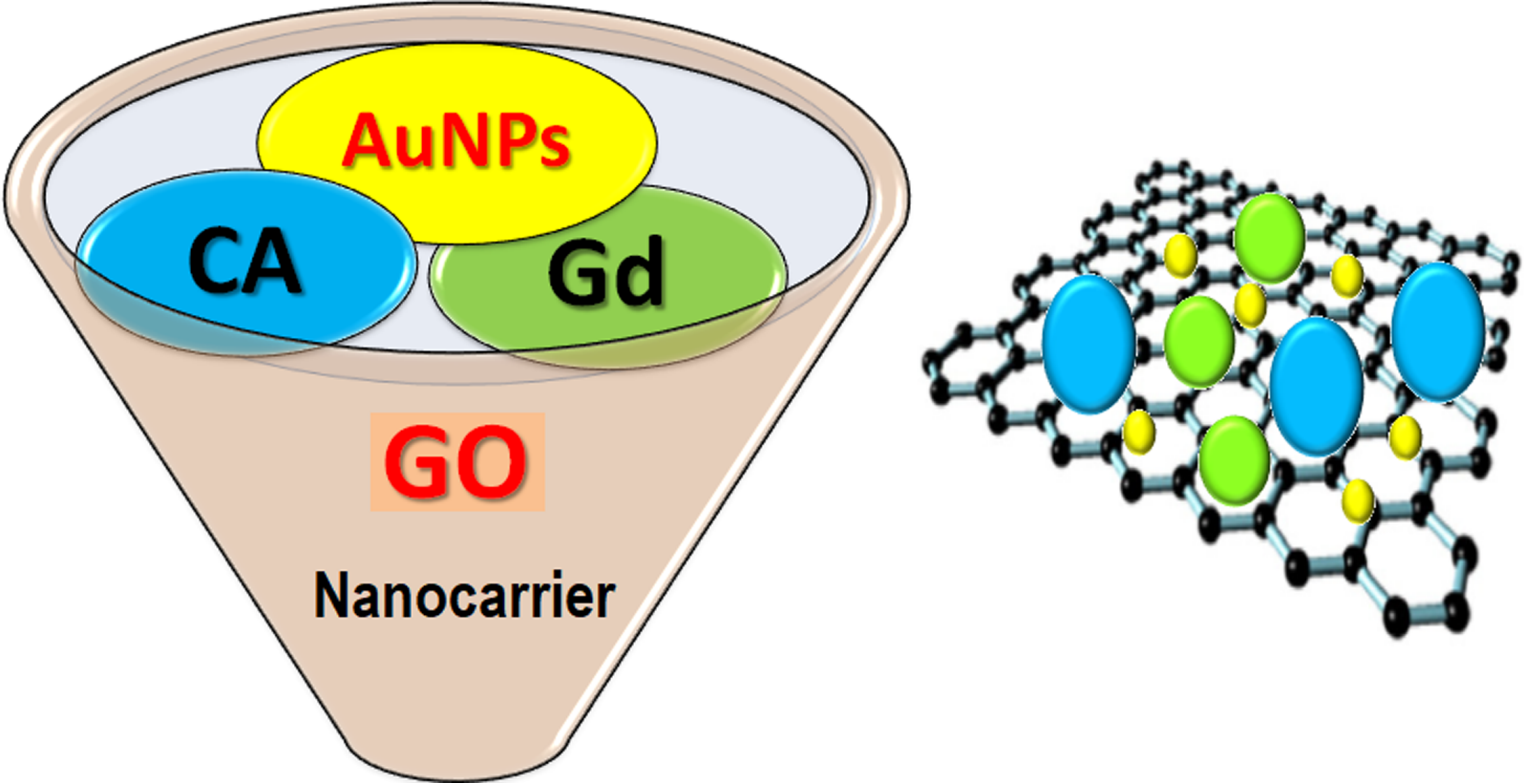
A bimodal theranostic nanodelivery system composition, as in BIT nanocomposite; diagnostic agents, gadolinium (green) and AuNPs (yellow) and the conjugated anticancer agent, chlorogenic acid (blue) are attached on a graphene sheet via hydrogen bonding and π─π interaction.

The pristine samples and the synthesized nanocomposites were characterized using various characterization techniques. The anticipated interaction between the *host*, GO nanosheets and the chlorogenic acid guest is via hydrogen bonding. The bonding is expected through the aromatic structured drug, which has abundant −OH groups. On the hand, GO nanosheets have–OH and COOH groups, which partake in the bond [9]. Another possible interaction is through the sp^2^-carbon sites which facilitate the π‒π stacking interactions with the Gd(NO_3_)_3_or even the drug, as presented in the schematic in Fig 1. The AuNPs can be seen to be adsorbed on the GO nanosheets surface to complete the BIT.

### X-ray diffraction

**Fig 2:**
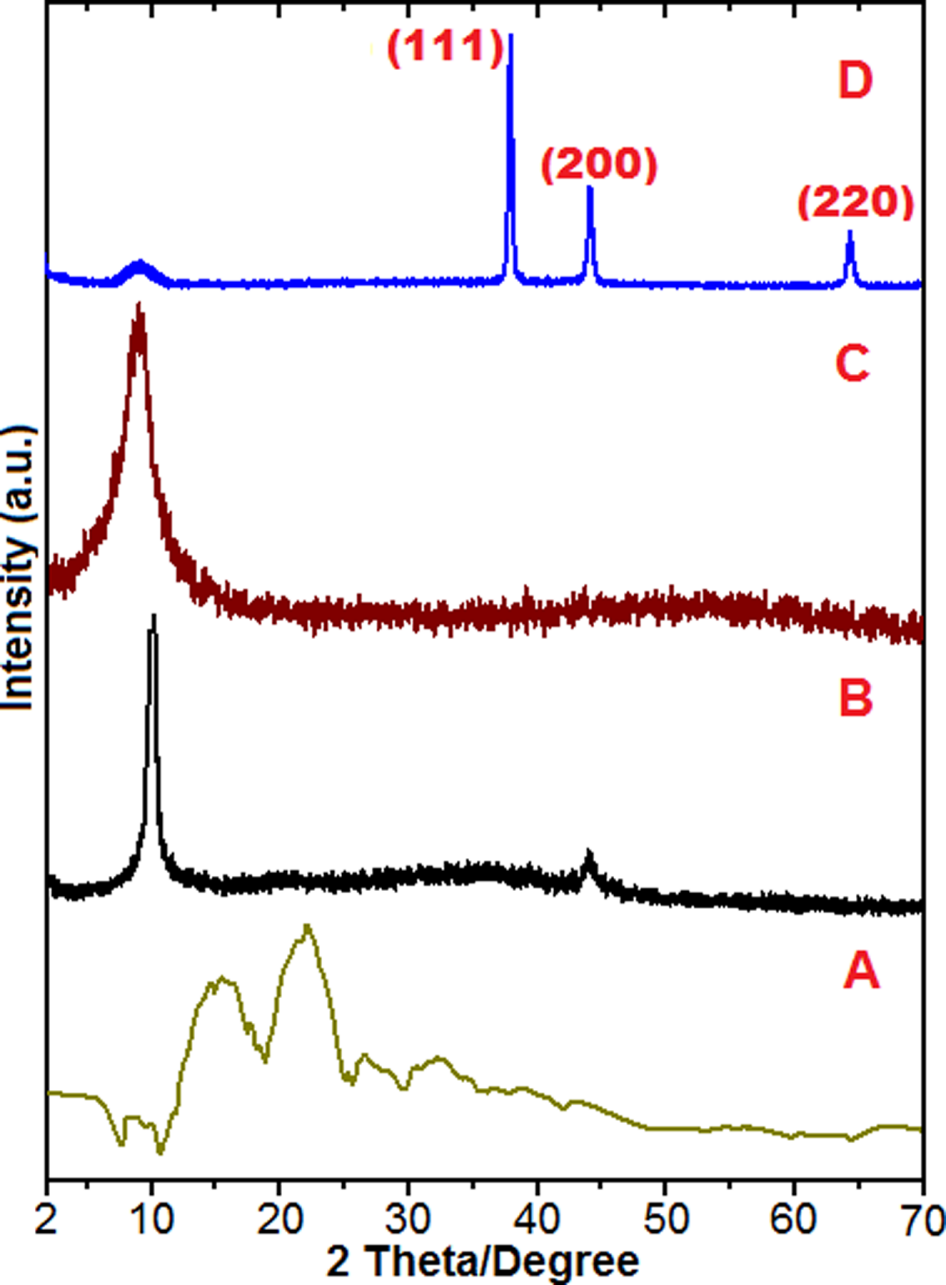
X-Ray diffraction patterns of pure chlorogenic acid (A), GO nanosheets (B), GO/Gd conjugated with chlorogenic acid (GOGCA) (C) and the bimodal theranostic nanodelivery system (BIT) composed of GOGCA-coated with AuNPs (D).

X-ray diffraction (XRD) is an important analysis tool in nanoscience research. XRD was employed to confirm the various stages of the GO‒based nanodelivery system development. As mentioned earlier, the research work started with the GO nanosheets synthesis and subsequently conjugation of the guest agents. Fig 2A‒2D shows the XRD patterns of the pure chlorogenic acid, pure GO, GOGCA nanohybrid and the BIT. The GO nanosheets diffractogram as obtained via the improved Hummer’s method can be seen to have one significant reflection at 2θ = 10° with d spacing of 8.5 Å, which is in agreement with the previously reported works [22], indicating the formation of the intended GO nanosheet (Fig 2B). Furthermore, the XRD patterns of the successive nanohybrids were obtained, starting with Gd and chlorogenic acid conjugated nanohybrid (GOGCA). The XRD pattern of the GOGCA also shows a single and broader reflection, which is slightly shifted to the lower 2θ angle as compared to the pure GO nanolayers (Fig 2C). The shift to the lower 2θ position suggests conjugation has taken place between the GO nanocarrier and the guest molecules [21]. The interaction is likely via π‒π stacking and hydrogen interactions. This is expected to influence the release profiles of the nanocomposite, which will be discussed later in the drug release studies. The aromatic chlorogenic acid has abundant OH groups to bond with epoxide (O) and COOH groups of GO nanosheets. The Gd is loaded via π‒π stacking bonding [23]. Although layered nanocarriers are capable on interlayer bonding and intercalation [15], the chlorogenic acid was π‒π and hydrogen bonded in this case, because no significant increase in the interlayer spacing was noticed after the drug loading. The reflections obtained for the AuNPs-coated nanohybrid that is the BIT diffractogram is shown in Fig 2D. Only a week reflection can be seen at slightly lower than 2θ = 10° position, where the GOGCA reflection is observed. All other reflections are of the AuNPs, which appears to have been surface adsorbed on the GO nanosheets. The 2θ positions at 38°, 45° and 65° are reflections of 111, 200 and 220, respectively of the face centered cubic (FCC) of AuNPs. The reflections match with the JCPDS reference file of AuNPs [24].

### Drug release and kinetics

**Fig 3:**
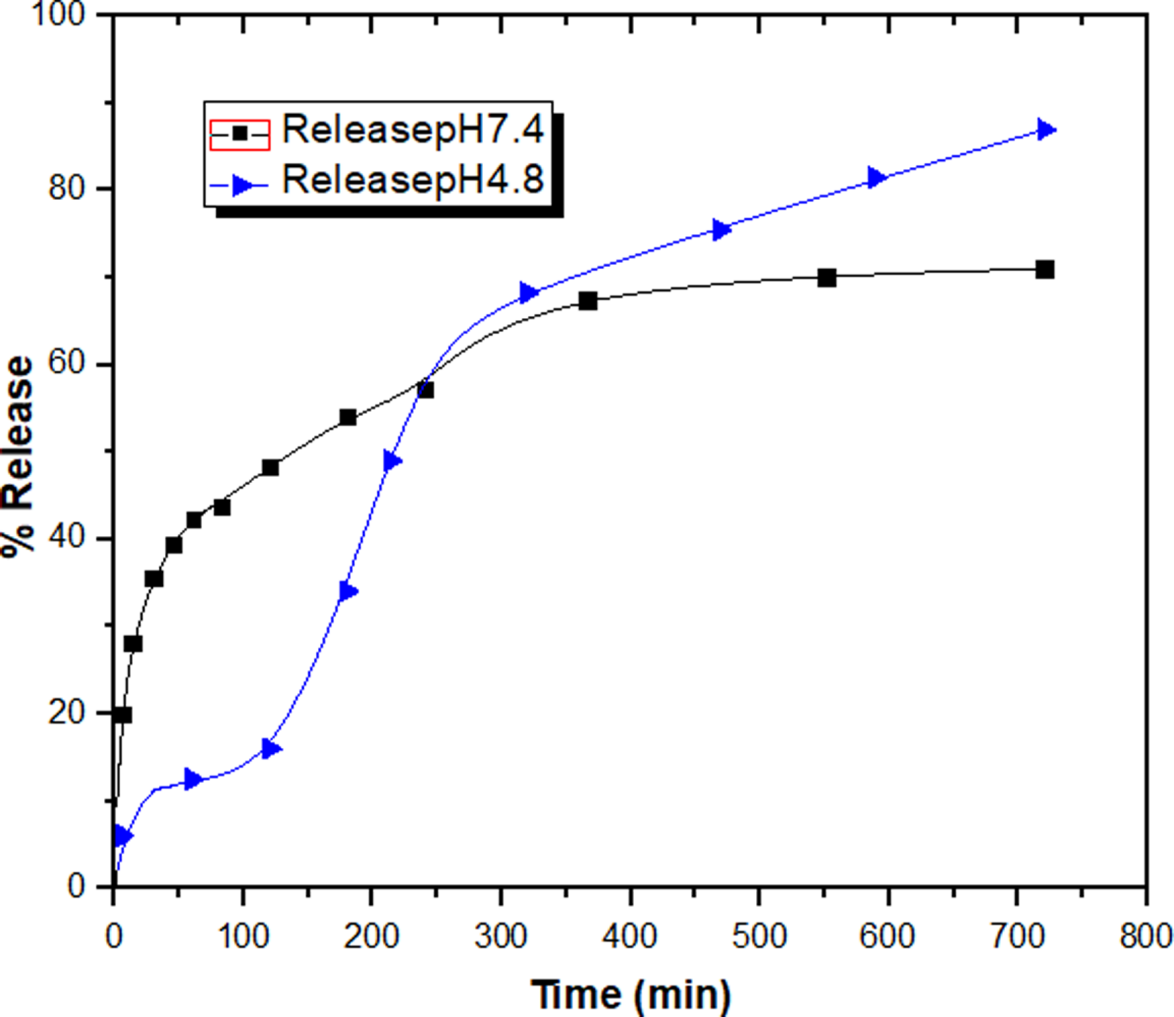
Chlorogenic acid release profiles from GOGCA nanocomposite in PBS media at pH 7.4 and 4.8.

The release of CA from the GOGCA nanohybrid was studied in PBS medium at pH 7.4 and 4.8. The manual approach was employed in the collection of the release media as described in the methodology section. Fig 3 depicts the release profiles of CA from GOGCA in the PBS media. As can be observed, the release period is from 0 to 700 min. Nevertheless, the actual release started at around 2 mins. The release in the acidic medium appears to be higher (up to 90%) than in the alkaline medium (70%). This may be due to higher hydrogen attraction in acidic condition which would weaken the bond between the OH bearing groups in the nanocomposite [23]. This process results in the gradual detachment of the chlorogenic acid from the GO nanosheets. As mentioned earlier, the GO is capable of hydrogen-bonding with the OH groups of chlorogenic acid. In addition, π‒π stacking interaction with the hydroxyl groups is also likely in the sp^2^ carbon atoms of the GO nanocarrier [25]. Nonetheless, the hydrogen bonding is more likely due to the structure of chlorogenic acid and GO. The GOGCA release profiles may indicate successful release of the anticancer agent in the tumor-sight and less release in the blood stream [26]. Our drug release profiles are higher than some of the previously reported GO-based drug delivery systems, including those conjugated with compatibilizers [27].

The drug kinetics was further studied with the drug release data to further understand the mode of the release. Three kinetic models were employed for the study, which are pseudo−first order (1) pseudo−second order (2) and parabolic diffusion (3). The equations are depicted below:

$$\left(\ln(q_{e}-q_{t}=\ln q_{t}-kt\right)$$ (1)

( tqt=1kqe2+tq) (2)

$$(1-M_{t}/M_{o})/\text{t}=kt^{-0.5}+bM_{t}q_{t}$$ (3)

**Fig 4:**
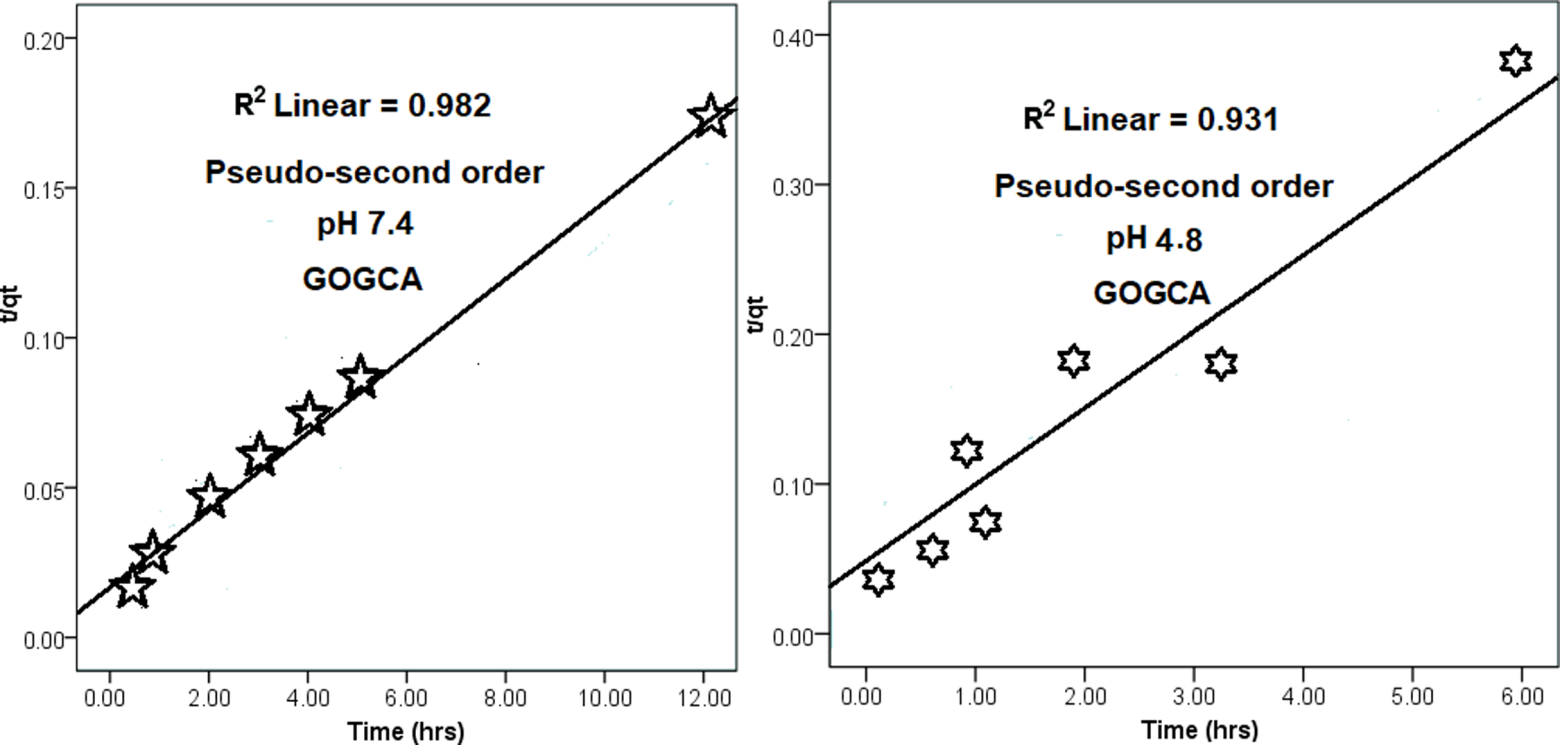
The data of chlorogenic acid release from the GOGCA nanocomposite at pH 7.4 and 4.8 media as fitted to pseudo−second order kinetic model.

The three models have different variables, for instance, $q_{e}$ and $q_{t}$ used in the pseudo-first order indicate the amounts of drug release at the equilibrium (e) and at time (t) thus, ($\ln(q_{e}$ –$\;{\;q}_{t})\;$is plotted against time, from which correlation coefficient (*R*^*2*^) value is derived. Whereas, only the  tqt is plotted against time to obtain the *R*^*2*^ value in the pseudo-second order. Lastly, the parabolic diffusion is deduced from the amounts of chlorogenic acid in the nanocarrier at release time t and 0, denoted as $M_{t}$ and$\;M_{o}$, respectively and k is the rate constant found in all three equations [28]. As obtained from the three models, the most suitable model for the release is the pseudo-second order with *R*^*2*^ value of 0.982 and 0.931, for release in pH 7.4 and 4.8 respectively, while the rate constant (k) is $1.7\;\times \;{\;10}^{-2}$ g/mg h for pH 7.4 and $5.1\;\times \;{\;10}^{-2}$ g/mg h for pH 4.8. The data of chlorogenic acid release from the nanocomposite is presented in Fig 4. Table 1 summarizes all the correlation coefficients (*R*^*2*^), percentage saturation (%), half-life [t_*1/2*_ (min)] and rate constant (k) of the three models for easy comparison.

**Table 1:** Kinetic models information deduced from correlation coefficients (*R*^*2*^), percentage saturation (%), half-life (t_*1/2*_) and rate constants (k) of chlorogenic acid release from GOGCA at pH 7.4 and 4.8.

Sample pH	*Correlation coefficients* *(R*^*2*^*)*			Percentage saturation (%)	t_*1/2*_ (min)	Rate constant (*k*)
	Pseudo-first order	Pseudo-second order	Parabolic diffusion			
7.4	0.916	0.982	0.936	$1.7\;\times \;{\;10}^{-2}$	70	40
4.8	0.868	0.931	0.850	$5.1\;\times \;{\;10}^{-2}$	90	50

### Raman spectroscopy studies

**Fig 5:**
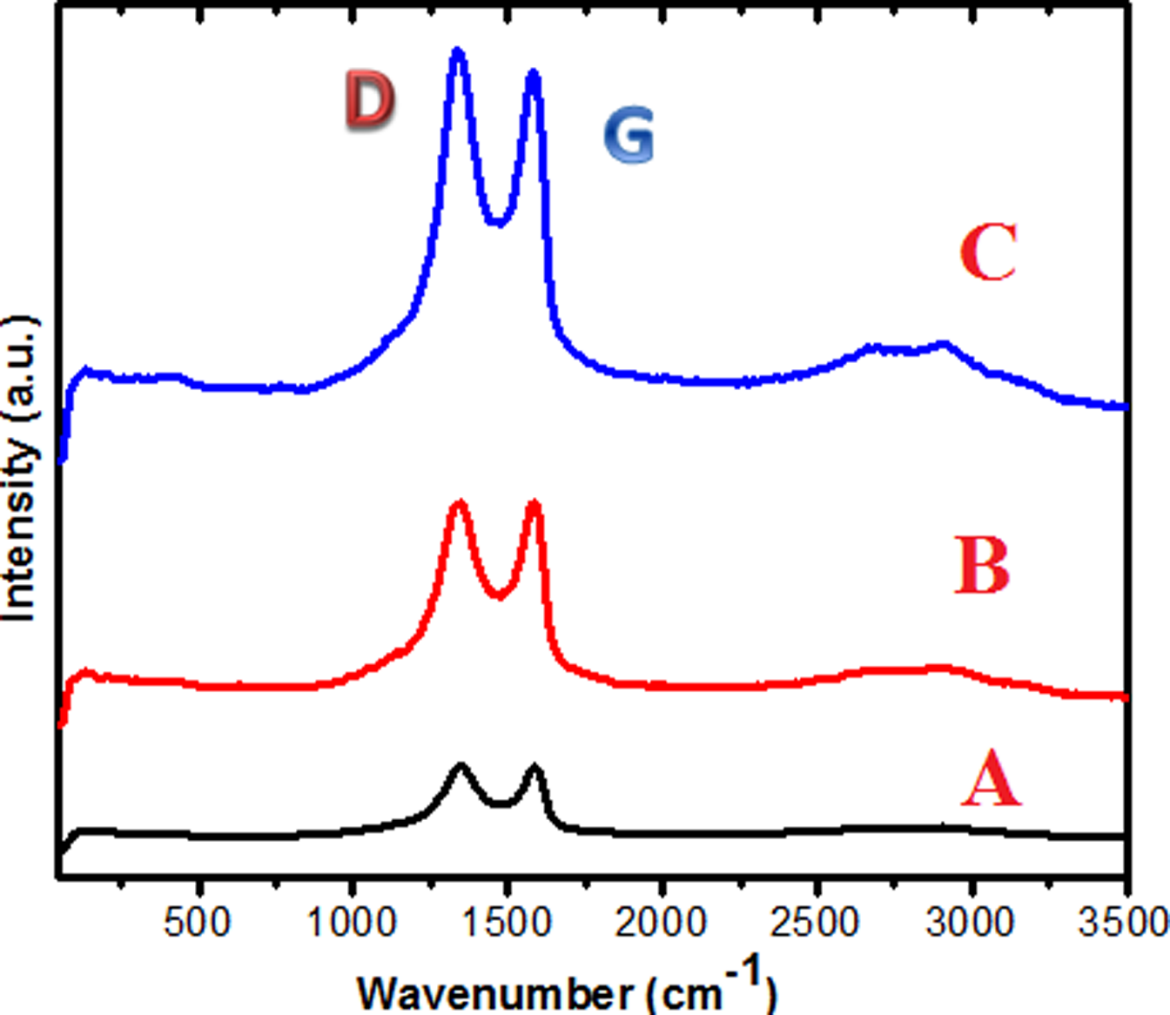
Raman spectra of GO nanosheets (A), GO/Gd conjugated with chlorogenic acid (GOGCA) (B) and the bimodal theranostic nanodelivery system [(BIT) GOGCA coated with AuNPs] (C).

Raman spectroscopy was employed to confirm the conjugation of the guest molecules on the GO nanosheets. The study is based on the degree of disorder, which is expected to increase upon successful functionalization or conjugation of the GO nanosheets. There are two major bands in the Raman spectrum for graphite-based materials; the disorder band (D) and the graphitic band (G). Fig 5 shows the Raman spectra of pure GO nanosheets, GOGCA and BIT, A−C respectively. The intensities of the D and G bands can be seen to be proportional to the conjugation activities on the GO nanosheets. For example, the intensities of the D and G bands in the pure GO are less than those of the nanohybrid functionalized with Gd and chlorogenic acid (GOGCA) in Fig 5B. Similarly, the D and G band intensities of the BIT are higher than those of GO and GOGCA. The reason for these variations is linked to the conjugation of chlorogenic acid with GO nanosheets as well as π‒π interactions with Gd sp^2^-carbon. In the BIT Raman spectrum (Fig 5C), the intensity of the disorder band (D) appears to be stronger than the graphitic band (G), which is understandably due to electrostatic interactions between GO nanosheets and positively charged AuNPs coated on the surface [25].

To buttress this assertion, ratio of the degree of disorder to graphitic in the nanocomposites was further analyzed. The ratio is deduced from the intensities of the D and G bands (I_D_/I_G_), which indicates the extent of disorder/functionalization of a graphitic material [29–31]. The GO nanosheets appear to have the least I_D_/I_G_ ratio (0.84), followed by the GOGCA at 0.86 and lastly the BIT with the highest (0.94). The steady rise in the values also confirms the sequential conjugation of the guest molecules. The analogy between the X-Ray diffractive patterns of the samples and the Raman shifts of the sample is quite obvious and confirm the formation of the BIT. Similar results were observed by Barahuie *et al*., 2017 who used GO nanosheets to conjugate other therapeutic agent for drug delivery application [21].

### Chemical interactions

**Fig 6:**
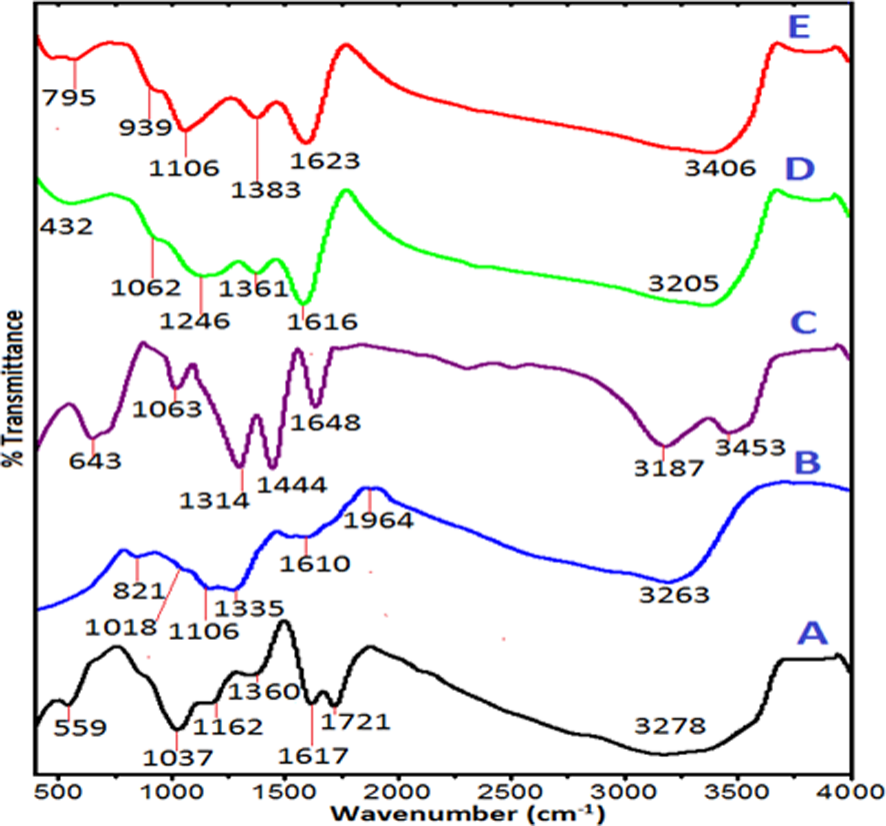
FTIR spectra of GO nanosheets (A), pure chlorogenic acid (B), ${Gd\left({NO}_{3}\right)}_{3}$ (C) GO/Gd conjugated with chlorogenic acid (GOGCA) (D) and the bimodal theranostic nanodelivery system [(BIT) GOGCA coated with AuNPs] (E).

The chemical interactions between the pure phases and the nanohybrids were studied with fourier-transformed infrared spectroscopy (FTIR). The spectra of the pure GO nanosheets, pure chlorogenic acid, GOGCA and BIT are presented in Fig 6A−6E. The spectrum in Fig 6A shows the absorption bands of GO nanolayers, starting with a broad and intense–OH stretching band at 3278 cm^−1^. The band is ascribed to the presence and bonding activity of the hydroxyl groups of the GO nanosheets [32]. The absorption band linked to the carbonyl and carboxylic acid groups can be seen at 1721 cm^−1^, in form of C = O stretching vibration, while the residue of graphite sp^2^ carbon can be seen at 1617 cm^−1^ as C = C bond [33].

The band at 1360$\;{cm}^{-1}$ is associated with COH [30]. The bands at 1162 and 1037 cm^−1^ are ascribed to C–O stretching vibrations. The OH bending vibration also appeared at 559 ${cm}^{-1}\;$due to the carboxylic group. In the chlorogenic acid FTIR spectrum (Fig 6B), the band at 3263 ${cm}^{-1}$ is for the OH stretching vibration [34], the C═O stretching vibration peak can be seen at 1964 ${cm}^{-1}$ and the C═C aromatic ring stretching band is at 1610${\;cm}^{-1}$ [35]. The bands at 1106 and 1018 ${cm}^{-1}$ are due to C─O stretching vibration and the band at 821 ${cm}^{-1}$ is assigned to CH bending vibration. Fig 6C depicts the Gd(NO_3_)_3_ spectrum, the O—H stretching vibration bands can be seen at 3453 and 3187$\;{cm}^{-1}$ [32], while the $H_{2}O$ bending vibration appeared at 1650$\;{cm}^{-1}$ [36]. The NO_3_-stretching vibration bands also appeared at 1444 and 1314$\;{cm}^{-1}$ [37]. The spectrum of the GOGCA nanohybrid is shown in Fig 6D. A shift in the positions of the absorption bands can be observed when compared to the pure phases, likewise some new bands are formed, which all can be due to new interactions between the GO nanosheet and the guest compounds. The C = O stretching vibration from carboxylic group of the GO at 1721 ${\;cm}^{-1}$ and the C = O stretching vibration of the chlorogenic acid at 1964${\;cm}^{-1}$ are all conspicuously missing in the GOGCA spectrum (Fig 6D), while a sharp and more defined C═C bond can be observed at 1616${\;cm}^{-1}$. The COH band has shifted to 1361$\;{cm}^{-1}$ and a new C─O stretching vibration can be seen at 1246$\;{cm}^{-1}$. The carboxylic group absorption bands in form of OH bending vibrations appeared at 432${\;cm}^{-1}$. The new absorptions bands and shifts are as a result of chemical interactions [20], mainly hydrogen bonding between GO/Gd and chlorogenic acid.

The absorption bands in the BIT spectrum in Fig 6E are almost identical with those of the GOGCA spectrum. Only slight shifts can be seen in the absorption bands due to presence of AuNPs at the surface. The FTIR spectra conform with the Raman shifts and the XRD diffractograms of the developed nanodelivery system, which all together indicate successive loadings of Gd, CA and AuNPs onto the GO nanosheets

### Transmission electron microscopy

**Fig 7:**
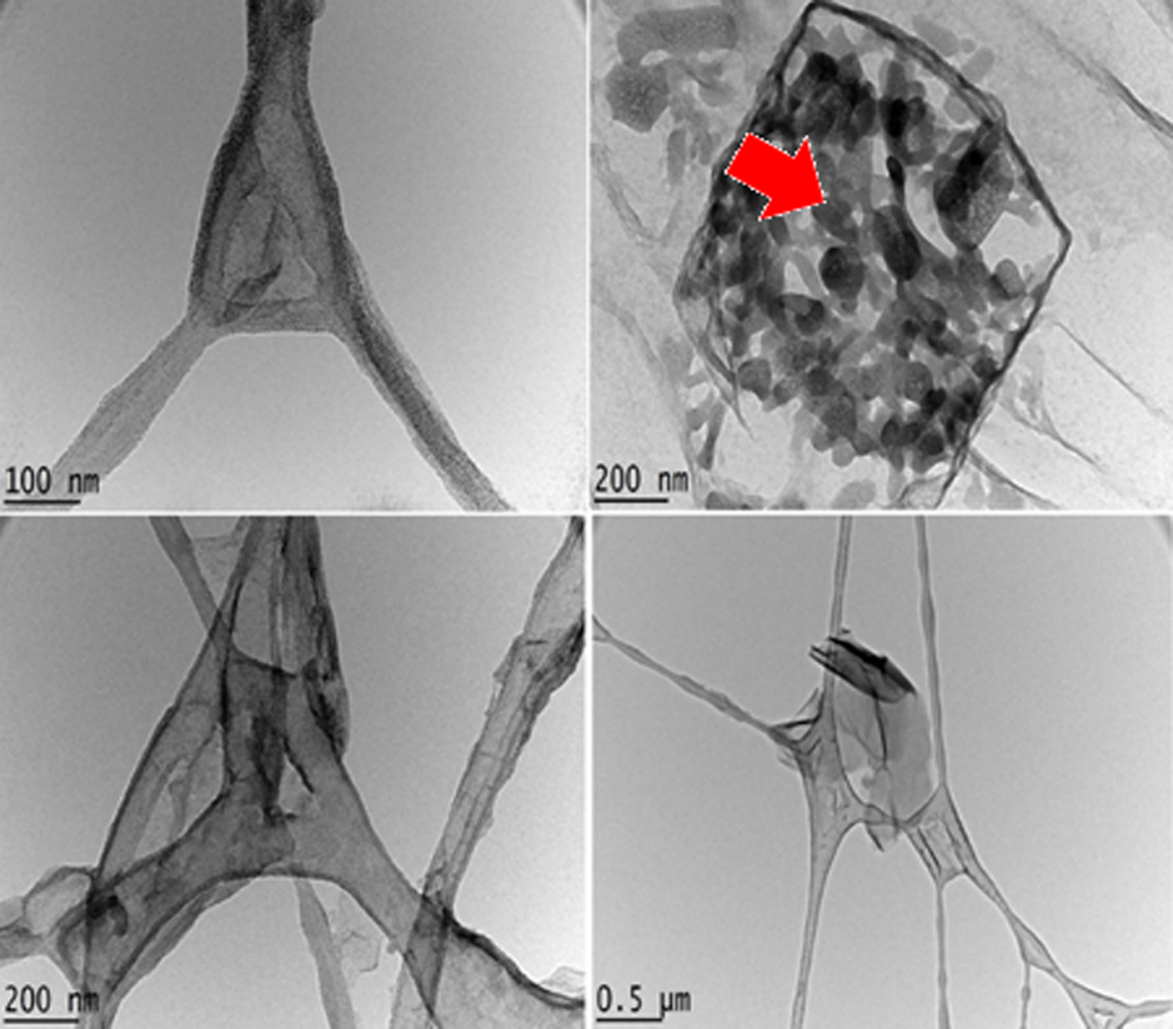
TEM micrographs of GO nanocarrier conjugated with chlorogenic acid and Gd (GOGCA) at different magnifications.

Transmission electron microscopy (TEM) was used to study the morphologies and shapes/sizes of the drug conjugated nanocomposite (GOGCA) as well as the AuNPs coated nanohybrid (BIT). Fig 7 depicts the morphology of GO conjugated with Gd and chlorogenic acid. The multiplayer GO nanosheets captured from different angles can be viewed in all the micrographs. The deposited chlorogenic acid can be seen in the micrographs with a red arrow pointing the area of the large deposition. This further affirms the XRD diffractogram of the GOGCA, which suggests conjugation of the chlorogenic acid and Gd.

**Fig 8:**
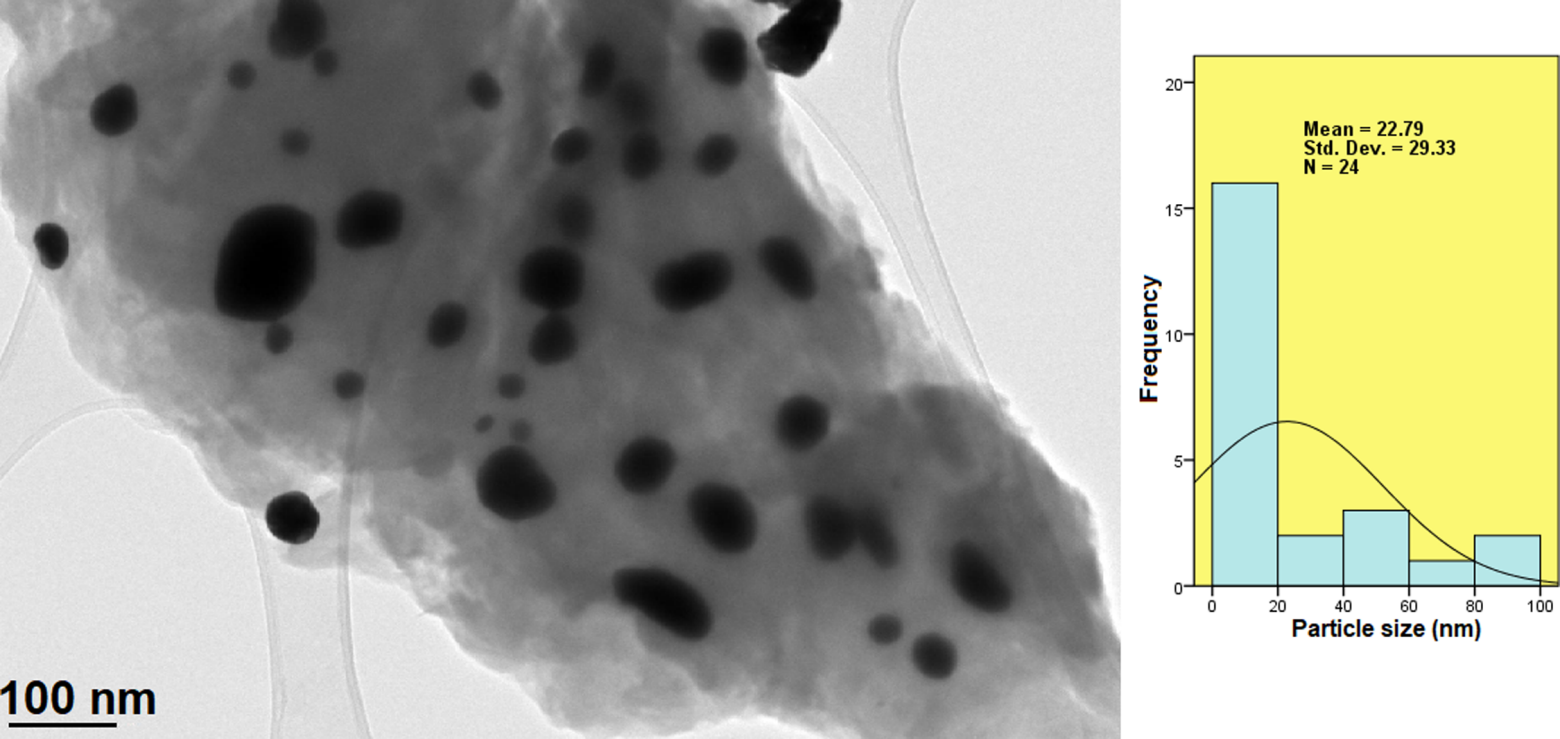
TEM micrographs of GO nanocarrier conjugated with chlorogenic acid and Gd (GOGCA) after AuNPs coating (BIT).

The micrograph of the AuNPs deposited on the GOGCA nanohybrid (BIT) is shown in Fig 8. The AuNPs appear mainly spherical in shape and distributed in different sizes. Nevertheless, almost all the nanoparticles are within the nano-range and a significant number are less than 20 nm in size, as shown in the size distribution histogram in Fig 8. This is also an affirmation of the XRD diffractogram of the BIT, which suggests adsorption of AuNPs on the GO surface via electrostatic interactions.

### Elemental composition studies

Inductively coupled plasma atomic emission spectrometry (ICP‒ES), carbon, hydrogen, nitrogen and sulphur (CHNS) and energy dispersive X-ray spectroscopy (EDS) were combined to analyze the composition of the GO nanocarrier and the developed BIT. The ICP‒ES analysis was used for the Au and Gd content estimation and CHNS analysis for the C and H content. Similarly, the EDS was used to determine the C, O, Gd and Au elements that are present in the nanocomposite. Table 2 presents the estimated values derived from the CHNS and ICP‒ES analyses. It has been established from the results of the characterization studies discussed in the previous sections, that conjugation between GO functional groups and chlorogenic acid has taken place. It is therefore expected for the number of C and H content to be higher in the developed BIT than in the pure GO nanosheets. From Table 2, the C and H percentages can be observed to have increased from 36.8 and 2.5 to 44.4 and 2.9, respectively, that is GO to BIT, respectively; the increase is an indication of conjugation of aromatic chlorogenic acid onto GO nanolayers.

**Table 2:** Elemental compositions (%) of GO nanosheets and the BIT nanohybrid.

Sample	C^1^	H^1^	Gd^2^	Au^2^
**GO**	38.6	2.5	-	-
**BIT**	44.4	2.9	0.8	0.9

^1^ CHNS analysis

^2^ ICP‒ES analysis

**Fig 9:**
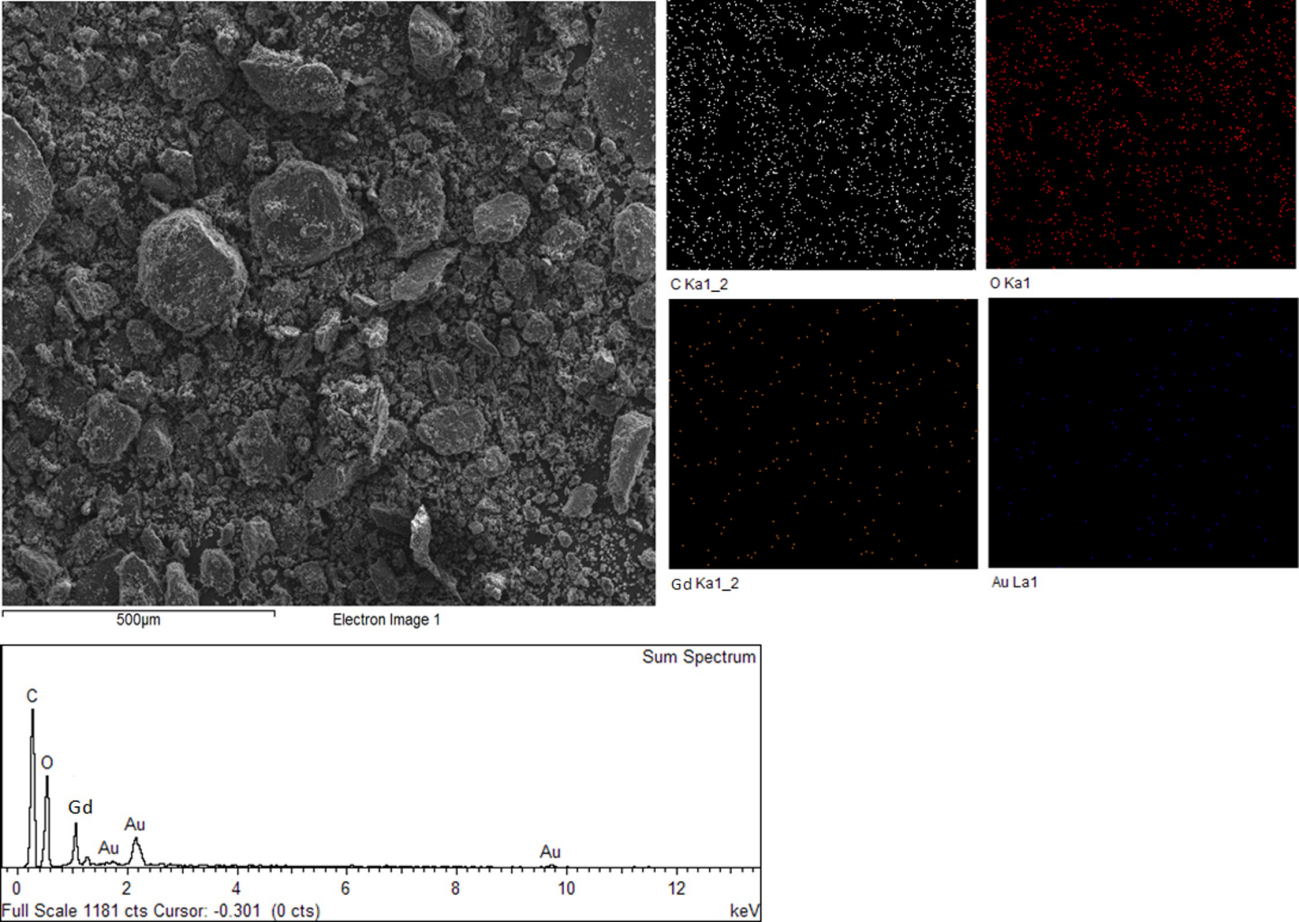
Energy dispersive X-ray spectroscopy mapping and spectrum of GO conjugated with chlorogenic acid and Gd, as well as after coating with AuNPs (BIT).

More so, the detections of Gd (0.8%) and Au (0.9%) in the BIT and their absence in the pristine GO, also confirms the electrostatic adsorption of the elements onto the GO nanosheets and subsequent formation of the BIT. In addition, the components of BIT have been further confirmed by the EDS results in Fig 9. Elemental mapping of the micrographs of the BIT shows the presence of C, O, Gd and Au, which have been outlined in Table 3.

**Table 3:** Elemental composition (%) of BIT nanohybrid as deduced from elemental mapping.

Element	App	Intensity	Weight (%)	Weight (%)	Atomic (%)
	Conc.	Corrn.		Sigma	
**C K**	66.30	0.9785	47.78	1.34	60.16
**O K**	34.25	0.6464	37.35	1.31	35.30
**Na K**	6.98	0.9444	5.21	0.34	3.43
**Gd K**	0.76	0.7800	0.68	0.16	0.43
**Au M**	9.97	0.7838	8.97	0.90	0.69
**Totals**			100.00		

The atomic percentage of C stands at 60.1 and O at 35.1. The high number of C and O atoms in the BIT is due to the carbon and oxygen-based structure of GO coupled with C and O content of the conjugated chlorogenic acid. The percentage atomic counts of Gd and Au can equally be seen at 0.43 and 0.69, respectively. The elemental composition studies complement the FTIR spectra of the pure phases and the nanocomposites, which all indicate sequential loading of guest molecules by the GO nanocarrier. Further, the absence of other elements in the mapping spectra also suggest the nanohybrids are considerably of high purity.

### Thermal gravimetric analysis

**Fig 10:**
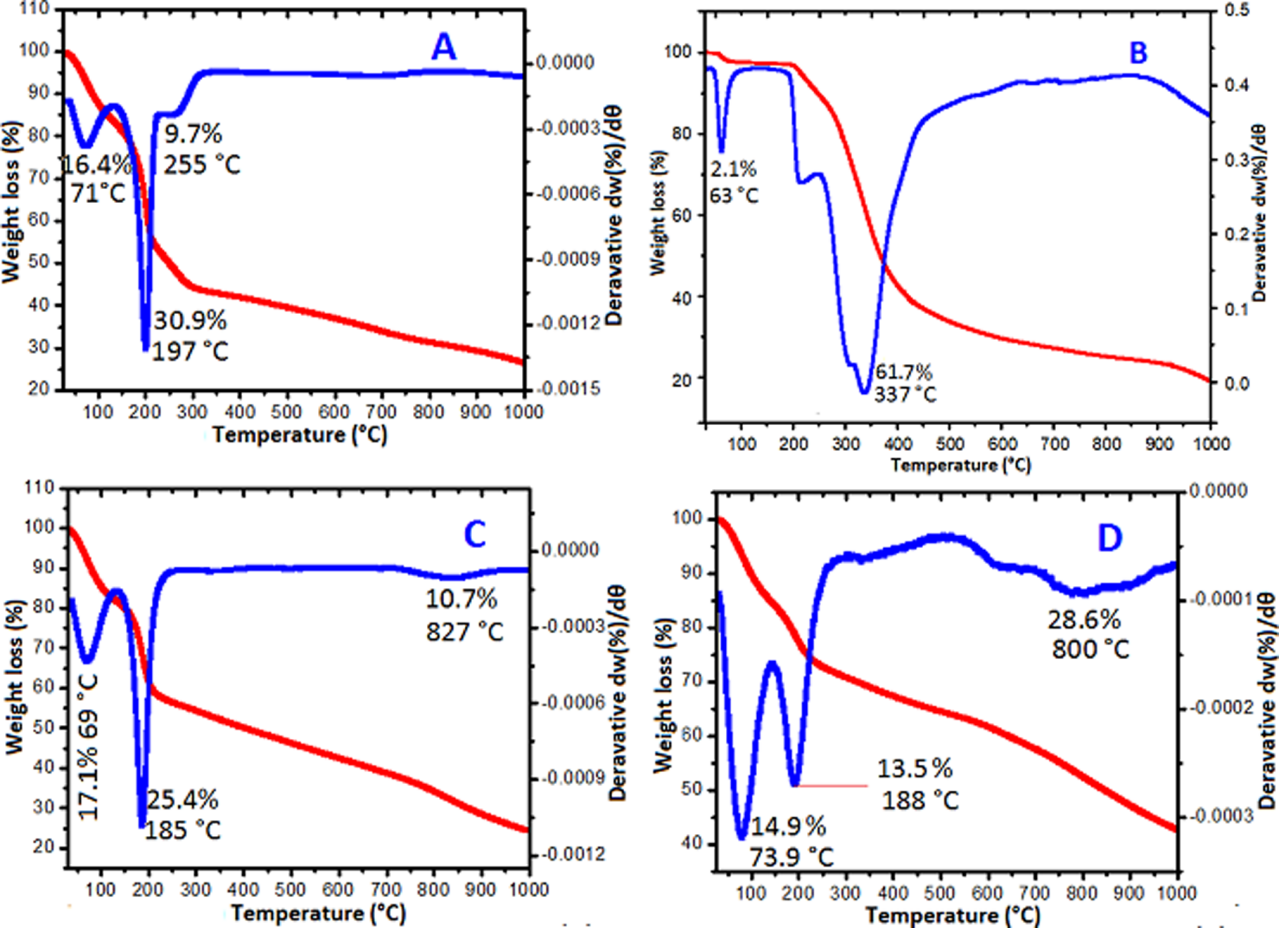
TGA thermograms of GO nanosheets (A), pure chlorogenic acid (B), GO/Gd conjugated with chlorogenic acid (GOGCA) (C) and the bimodal theranostic nanodelivery system [(BIT) GOGCA coated with AuNPs] (D).

Thermal analysis was further used to authenticate the composition of the nanohybrid systems. Thermal gravimetric analysis (TGA) and its derivative (DTG) were applied in this work for this purpose. The thermograms of the pure phases were first collected and subsequently the thermograms of the nanocomposites. Fig 10A represents the thermal decompositions of the pure GO nanolayers as obtained via the improved Hummer’s method. Three decompositions can be seen in the GO thermogram, which begins with 16.4% weight loss due to physically-adsorbed water at 71°C. The second and significant decomposition at 30.4% is ascribed to the reduction that occurred due to heating, which has broken down the bonds between graphene and oxygen groups. While the decomposition at 255°C (9.7%) could be from the residue of ash formed from GO reduction [38].

Fig 10B depicts the pure chlorogenic acid thermogram. There are two major thermal activities, one at 63°C which has weight loss of 2.1%. The decoposition is linked to early removal of moisture. The second and major weight loss is at 337°C with a substantial mass loss (61.7%), which isas a result of chlorogenic acid decomposition and an indirect combustion [39]. The thermogram of the GO/Gd conjugated with chlorogenic acid (GOGCA) nanocomposite is shown in Fig 10C. The pattern of decomposition is similar of that of the pure GO nanocarrier. The decomposition at 69°C, which has 17.1% weight loss is due to removal of water. The decomposition at 185°C and 25% weight loss is associated with GO decomposition, which appeared in a slightly higher temperature in the GO thermogram. The last decomposition at 827°C (10.7%) is likely due to decomposition of hydrogen bond between GO and chlorogenic acid. The high temperature decomposition after chlorogenic acid conjugation indicates thermal stability improvement of the CA. It also confirms the formation of the GOGCA nanohybrid. Further, the thermogram of the BIT, that is after AuNPs surface coating is shown in Fig 10D. The removal of water and GO decompositions can be seen at 73 and 188°C, with 14.9 and 13.5% mass loss, respectively. The major decomposition is at 800°C, which has a weight loss of 28.6%; this is presumably due to simultaneous decomposition of GO-chlorogenic acid bonds and the surface AuNPs. The thermal events are summarized in Table 4. The decomposition temperature range (T_range_), the maximum peak temperature (T_max_) and the change in mass (∆m) are the key thermal properties that are associated with the pure phases and the developed nanohybrids, which are all presented in the Table.

**Table 4:** Decomposition temperature range (T_range_) maximum peak temperature (T_max_) and change in mass (∆m).

Sample	T_range_ (°C)	T_max_ (°C)	Δm (% *w/w*)
**GO (A)**	71‒255	197	57
**Chlorogenic acid (B)**	63‒337	337	64
**GOGCA (C)**	69‒827	185	53
**BIT (D)**	73‒800	800	57

### Cytotoxicity studies

The extent of toxicity of the BIT as a theranostic agent was tested with standard fibroblast cell line (3T3) and human liver hepatocellular carcinoma cell line (HepG2). For the purpose of efficacy evaluation, both the pure GO nanolayers and the pure chlorogenic acid were included in the cytotoxicity studies. The three samples were prepared into different concentrations, 0.0, 1.6, 3.1, 6.3, 12.5, 25.0, 50.0 and 100.0 μg/mL and loaded into the HepG2 and 3T3 cell lines.

**Fig 11:**
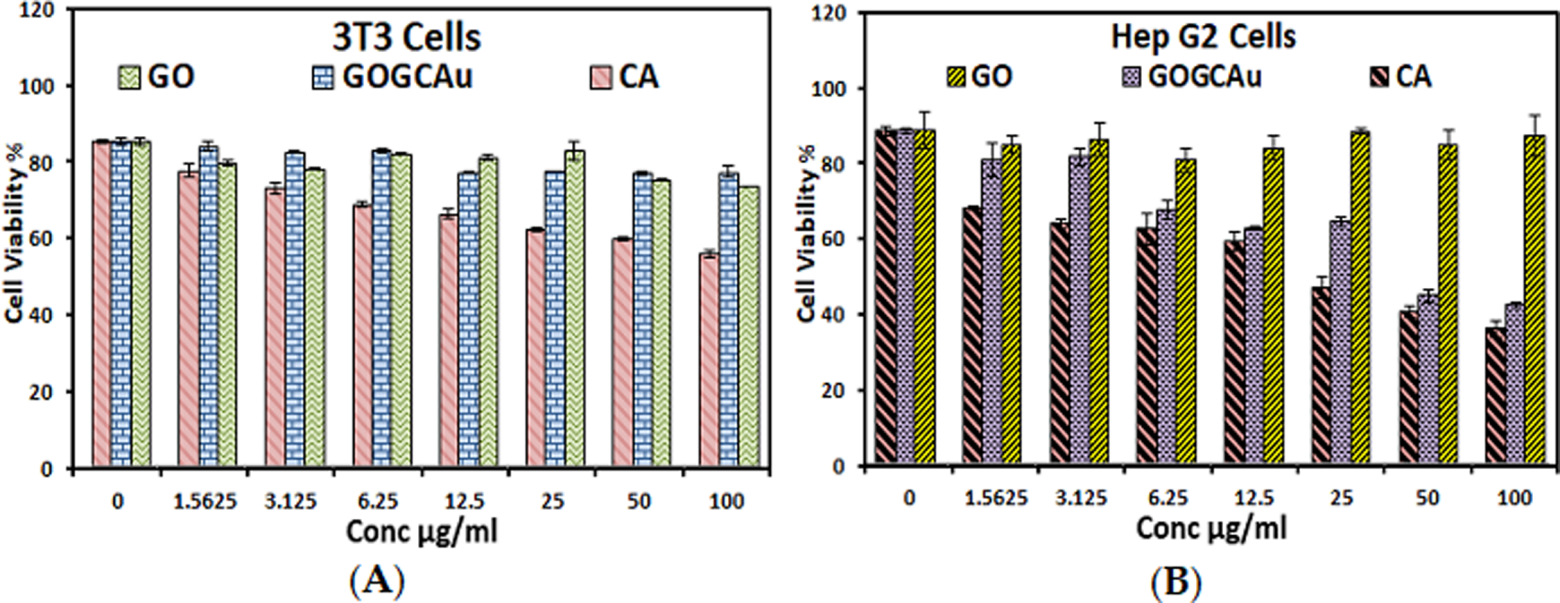
(A) Cytotoxicity results of fibroblast cell lines (3T3) dosed with pure GO nanosheets, pure chlorogenic acid and GO/Gd nanocarrier loaded with chlorogenic acid and coated with gold nanoparticles (BIT) and (B) Cytotoxicity results of human liver hepatocellular carcinoma cell lines (HepG2) dosed with pure GO nanosheets, pure chlorogenic acid and GO/Gd nanocarrier loaded with chlorogenic acid and coated with gold nanoparticles (BIT).

The inhibition zones of the cells after GO, chlorogenic acid and BIT loading were calculated and are presented in histogram form in Fig 11. From the histogram chart and the growth of the 3T3 cells in Fig 11A, it is apparent the non-toxicity of all the three samples, since all the cells have grown above average (50% cell viability) even at 100.0 μg/mL dose. This suggests the developed BIT as a relatively safe theranostic agent for cancer treatment.

Fig 11B depicts the toxicity studies of GO, chlorogenic acid and the BIT against the HepG2 cancer cells. From the growth of the cells in the GO doped cells at all concentrations, it can be concluded that the GO nanosheets are nontherapeutic towards the cancer cells. This is not peculiar to HepG2 cells alone as other cancer cell lines have been reported not to be susceptible to GO [11, 21, 40]. However, the growth of the cells is inhibited (below average) in the pure chlorogenic acid and the BIT‒loaded cells, especially in the 50.0 and 100.0 μg/mL doses. The cellular uptake of the BIT through the clathrin-mediated pathway [41] is the highest in the 50.0 and 100.0 μg/mL doses, which leads to more HepG2 endocytosis. This suggests the anticancer efficacy of BIT in the aforementioned concentrations and affirms its suitability as theranostic agents.

### Magnetic resonance imaging analysis

**Fig 12:**
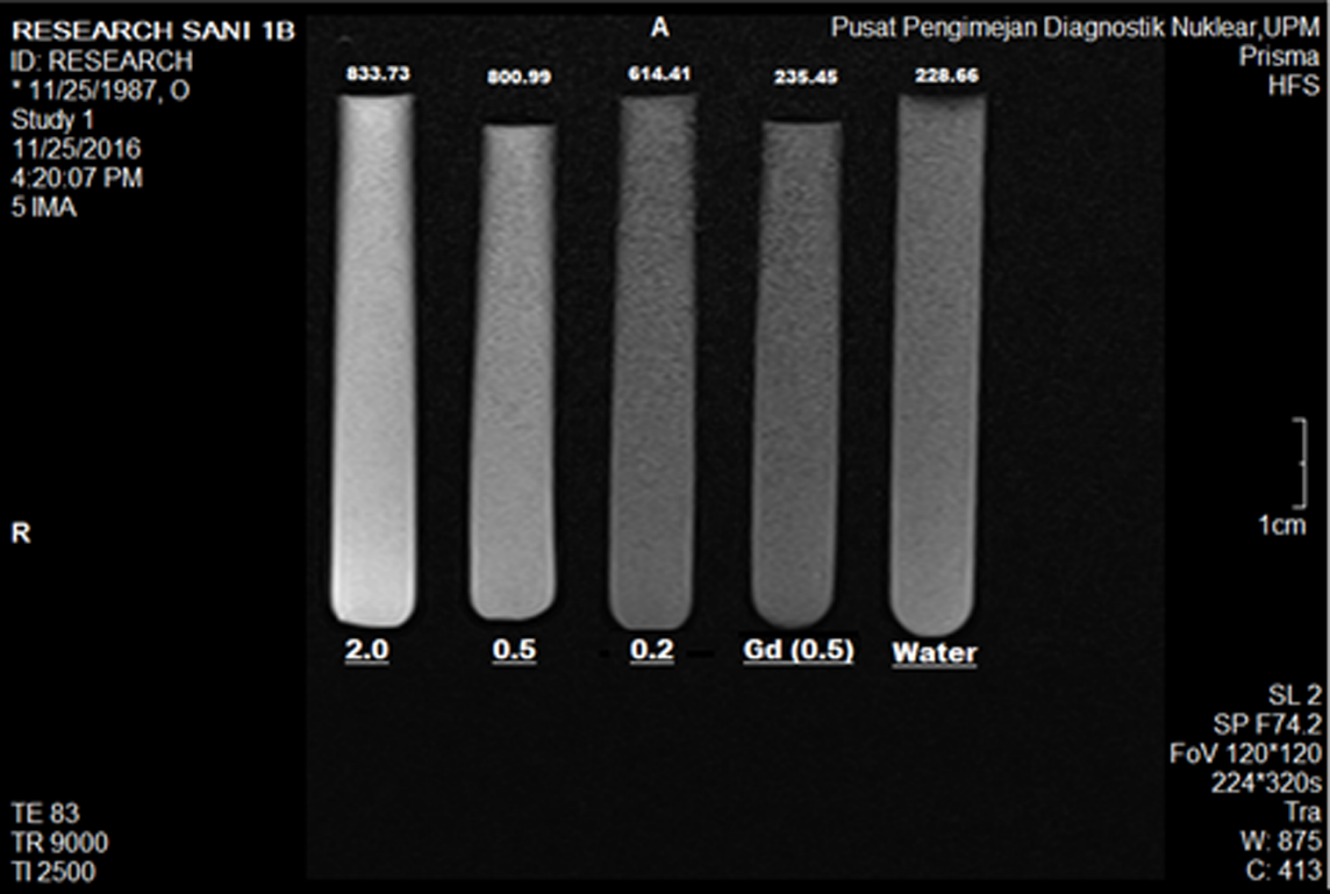
T1−weighted image of GO/Gd nanocarrier loaded with chlorogenic acid and coated with gold nanoparticles (BIT) at different Gd^3+^ concentrations (2.0, 0.5 and 0.2 *w*/*v*), 0.5 (Gd *w*/*v*) and water reference attained from a Prisma 3−Tesla MRI.

The theranostic modality of the BIT was further analyzed for contrast enhancement in the diagnostic component. The test was conducted on a magnetic resonance imaging (MRI) instrument, using 2.0, 0.5 and 0.2 *w*/*v* aqueous concentrations of the BIT with Gd^3+^ as the active agent and Gd(NO_3_)_3_ (0.5 *w*/*v*) and water as references. The contrasts/brightness of the BIT samples in the T1−weighted image can be observed to be increasing in order of the active agent concentration (Fig 12). The measured mean intensities of the individual tubes also confirm the apparent contrast enhancement. The intensities of the samples in the T1−weighted image increases in the same pattern as the contrast, with the 2.0 concentration having the highest signal intensity (833.73), followed by the 0.5 (800.99) and 0.2 (614.41) concentrations. The signal intensities are higher than the pure Gd at 0.5 *w*/*v* (235.45) and water (228.66). Similar observation has previously been reported by Usman *et al*., 2017 [20], where MRI signal was increased by the mixture of Gd and AuNPs. The increment is presumably due to the increased interactions between the ${Gd}^{3+}$ ions/AuNPs within GO nanosheets that result in a rise in the surface area at the GO surface and movement of water molecules. This can be understood from the concept of supramolecular arrangement of the GO-Gd/CA structure that is facilitated by hydrogen-boding interactions of the ‒OH and ‒COOH groups and the aqueous environment [2, 3], as well as electrostatic interactions with surface AuNPs, which is influenced by the high surface of area of the nanoparticles [20, 42]. The effect shortens and reduces the relaxivity of the MRI signal, thus improves the longitudinal relaxation time (T1 signal) [43, 44]. The results complete the diagnostic component of the nanocomposite and signify the developed BIT as a potential comprehensive future anticancer agent.

## Conclusion

An MRI focused theranostic nanohybrid system comprised of both anticancer and contrast agents have been developed in this work. The agents were loaded concurrently on GO nanosheets for the purpose of diagnosis and treatment. The experiment began with the preparation of GO nanolayers and electrostatic doping of Gd and hydrogen bonding of chlorogenic acid, as named GOGCA. Subsequently, the GOGCA was used as a base to adsorb AuNPs on the surface via electrostatic interactions to form BIT. About 90% of the chlorogenic acid was released from GOGCA under acidic cancer pH, which suggests high delivery in the cancer location. The efficacy of the BIT equally supports the release profile, which showed inhibited growth of HepG2 cells at 50 and 100 μg/mL concentrations, while nontoxic to normal 3T3 cells, with around 90% growth across all the BIT concentrations. BIT was observed to have increased the contrast of the T1‒weighted image tested with an MRI. The BIT interestingly showed higher signal than the conventional MRI contrast agent (Gd(NO_3_)_3_). Our in *vitro* preliminary results are promising and could serve as a guide in search of a holistic cancer medication in the future. We recommend animal studies on the BIT as a way forward.

## Supporting information

S1 Fig (XLSX)

S2 Fig (XLSX)

S3 Fig (OPJ)

S1 Text (TXT)
